# The Impact of Estrogens and Their Receptors on Immunity and Inflammation during Infection

**DOI:** 10.3390/cancers14040909

**Published:** 2022-02-12

**Authors:** Alfred T. Harding, Nicholas S. Heaton

**Affiliations:** 1 Institute for Medical Engineering and Sciences, Massachusetts Institute of Technology, Cambridge, MA 02142, USA; alfredth@mit.edu; 2 Department of Molecular Genetics and Microbiology, Duke University School of Medicine, Durham, NC 27710, USA; 3 Duke Human Vaccine Institute, Duke University School of Medicine, Durham, NC 27710, USA

**Keywords:** estrogen, sex hormones, inflammation, infection

## Abstract

**Simple Summary:**

Human health is significantly affected by microbial infections. One of the largest determinants of the outcomes of such infections is the host immune response. Too weak of a response can lead to enhanced spread by the pathogen, while an overstimulated response can lead to immune-induced tissue damage. Thus, to effectively treat infected individuals, it is critical to understand the regulators that control inflammatory responses. Recently, it has become widely accepted that estrogens, a class of sex hormones, are capable of dramatically altering the responses of host cells to microbes. In this review, we discuss how estrogens change the host immune response, as well as how these changes can alter the outcome of the infection for the individual.

**Abstract:**

Sex hormones, such as estrogen and testosterone, are steroid compounds with well-characterized effects on the coordination and development of vertebrate reproductive systems. Since their discovery, however, it has become clear that these “sex hormones” also regulate/influence a broad range of biological functions. In this review, we will summarize some current findings on how estrogens interact with and regulate inflammation and immunity. Specifically, we will focus on describing the mechanisms by which estrogens alter immune pathway activation, the impact of these changes during infection and the development of long-term immunity, and how different types of estrogens and their respective concentrations mediate these outcomes.

## 1. Introduction

Despite advances in medicine, infectious diseases remain one of the leading causes of human death globally [1]. These infections are caused by diverse organisms that infect a variety of tissues and organ systems. An essential line of defense in combating these invaders is our immune system, which works to both identify the pathogen and effectively clear it from the body. While it is critical that inflammation be rapidly activated during the early stages of infection to limit the spread of a microbe, it is well appreciated that overstimulation of the immune response can lead to host tissue damage and even death [2]. Interestingly, it has been reported for decades across a range of organisms that host sex alters the immune response to and mortality rate from a variety of infections. These data implicate sex hormones as potential regulators of antimicrobial responses [3,4,5,6,7,8].

While estrogens were traditionally thought to primarily regulate the female reproduction system, previous studies have demonstrated that estrogenic activity can regulate many pathways in the body, including immune responses [9,10,11]. In general, it is now appreciated that estrogens, and in particular E2, can control the proinflammatory signals/pathways of the immune system [12,13,14]. ERα and GPER1, two of the three known estrogen receptors that enact the observed effects of estrogens, are commonly associated with anti-inflammatory phenotypes [14,15,16,17,18]. Reports concerning the third receptor, ERβ, are not as consistent; some describe an anti-inflammatory role similar to ERα and GPER1 [19,20], while others indicate an increased ratio of ERβ is associated with proinflammatory signatures [21,22]. Thus, it is likely that reported differences in the effects of estrogen on the immune system are due to variations in receptor expression in cell types and during different physiological states. This review will highlight some of what we know regarding the effects of estrogens on the immune system, with a specific focus on their impact in the context of infection and immunity to different pathogens.

## 2. Estrogens and Their Mechanisms of Signaling

**Figure 1:**
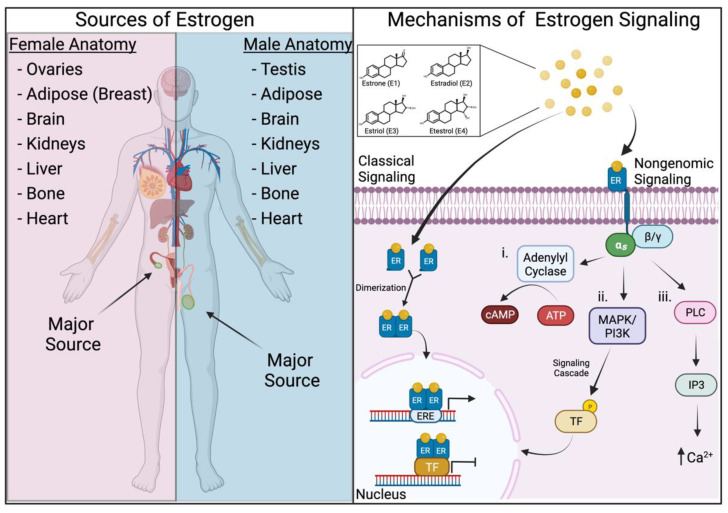
A summary of the sources of estrogen production in the body (**left**) and schematic diagram of both “classical” and “nongenomic” estrogen signaling pathways (**right**). Abbreviations: ATP: adenosine 5’-triphosphate, cAMP: cyclic adenosine monophosphate, ER: estrogen receptor, ERE: estrogen-responsive element, TF: transcription factor, α_s_: alpha subunit, β/γ: beta/gamma subunit, MAPK: mitogen-activated protein kinase, PI3K: phosphoinositide 3-kinase, PLC: phosphoinositide-specific phospholipase C, and IP3: inositol triphosphate. Created with Biorender.com, accessed on 9 December 2021.

Estrogens are a class of sex hormones whose function has been well characterized in the regulation of the development and function of the female reproduction system [23]. There are four forms of endogenous estrogen that are responsible for the effects attributed to this hormone: estrone (E1), estradiol (E2), estriol (E3), and etestrol (E4), with estradiol being both the most prevalent and potent estrogen prior to menopause [24]. These hormones are synthesized using cholesterol as the base sterol, with the enzyme aromatase converting them into estrogens [25]. In premenopausal females, these estrogens are primarily synthesized in the ovaries, as well as in the placenta during pregnancy; after menopause, ovarian-produced estrogen levels sharply decline and the majority of remaining estrogens are synthesized in secondary tissues such as the brain, kidneys, bones, skin, and adipose tissues (Figure 1, left) [24,26,27]. In males, the single largest producer of estrogen is the testis; however, estrogen produced from secondary tissues accounts for a much greater percentage of total estrogen in males as compared to females (Figure 1, left) [28].

The effects of estrogens are primarily enacted by their binding with estrogen receptors (ERs) via two distinct mechanisms. The classical mechanism involves estrogen diffusing into the cell and binding to the ER in the nucleus to stimulate the activation or repression of specific genes [29] (Figure 1, right). Additionally, estrogens can stimulate cells more rapidly through “nongenomic” mechanisms by binding to ERs localized to the plasma membrane or endoplasmic reticulum to stimulate immediate responses such as changes in Ca^2+^ levels or kinase activity [30,31] (Figure 1, right). Alongside receptor localization, the downstream functions of estrogens are dictated by which receptor is bound. Currently, it is appreciated that there are three estrogen receptors: estrogen receptor alpha (ERα), estrogen receptor beta (ERβ), and guanine nucleotide-binding protein-coupled estrogen receptor 1 (GPER1/GPR30) [32,33,34]. Even further tailoring of estrogenic effects is accomplished by distinct splicing variations and expression patterns of these ERs in tissues, creating the opportunity for a variety of estrogen-induced responses across the body [35,36,37,38].

## 3. Influence of Estrogens during Viral Infection and Pathogenesis

### 3.1. Estrogens and Their Receptors Regulate Innate Immune Responses to Viral Infection

After infecting a cell, viruses are sensed by cellular pattern recognition receptors, such as toll-like receptors (TLRs), cGAS/STING, and RIG-I-like receptors (RLRs), that initiate antiviral immune responses. This cascade of signaling events activates interferon-regulatory factors (IRFs) and nuclear factor-κB (NF-κB) to induce the expression of type-I interferons (IFNs), interferon α (IFNα) and interferon β (IFNβ), as well as other proinflammatory cytokines [39]. Type-I IFNs are an initial line of antiviral defense that can signal both via autocrine and paracrine mechanisms after secretion to induce the expression of interferon-stimulated genes (ISGs), which are responsible for antiviral activities inside the infected host cell.

Estrogens have been shown to regulate the type-I IFN response through several potential mechanisms, subsequently impacting the outcome of viral infection. One of the most common mechanisms is by preventing cells from producing type-I IFNs altogether. It has been shown that this can occur via direct interactions between ERα, GPER1, and NF-κB [16,40], by ERβ occupying NF-κB transcriptional sites, or through ERs inducing the expression of the NF-κB inhibitor Iκβα [19,41]. Additionally, we have recently shown that estrogens, especially E2, can activate GPER1 to directly suppress IFN signaling [14]. While the mechanism of this suppression is yet to be elucidated, it highlights that both the production of and response to IFNs, two early steps in the antiviral immune response, can be impacted by estrogens.

While type-I IFNs are primarily responsible for early activation of antiviral immunity and inflammation, inflammatory cytokines/chemokines play an important role in the recruitment and activation of immune cells, such as macrophages and neutrophils. The production of these cytokines, which drastically alter the immune state in the body, is also a result of the activation of NF-κB [42]. Correspondingly, it has been shown that increases in estrogen lead to the suppression of many proinflammatory cytokines via blocking NF-κB signaling [6,43,44,45]. While there is a large body of literature demonstrating how estrogens suppress inflammatory responses, some groups have reported a proinflammatory, or even a dual pro- and anti-inflammatory, effect depending on the specific cytokine/cell type studied [46]. E2 treatment has been reported to enhance IFN-γ, a type-II interferon, production [47]. Type-II IFNs, unlike type-I IFNs, are predominantly produced by natural killer (NK) cells after their detection of various cytokines, including type-I IFNs, IL-12, IL-15, and IL-18, which results in phosphorylation of STAT4 [48]. Differences in the mechanisms regulating and cell types producing type-II IFNs may explain why estrogens exhibit a different regulatory effect when compared to type-I IFNs.

### 3.2. Estrogens and Their Receptors Modulate Viral Infection Severity

High levels of the replication of some viruses induces significant immune activation, eventually leading to uncontrolled inflammation. This signaling, often termed a “cytokine storm”, is associated with severe disease and can cause further damage to the host leading to poor infection outcomes [49]. As potentially anti-inflammatory hormones, it follows that high levels of estrogens are associated with better outcomes following a variety of viral infections that can cause severe inflammatory states. Work by the Klein lab has shown that outcomes from influenza infection, a respiratory virus capable of inducing a cytokine storm phenotype, are improved when estrogens levels are increased [6,50,51]. Logically, this has been linked to the ability of estrogens to reduce the harmful overexpression of proinflammatory cytokines, creating a more balanced immune response that is able to clear the virus without severely damaging the host. This work agrees with a study in a murine pregnancy model of influenza infection where heightened estrogen levels during pregnancy suppressed inflammatory markers during influenza infection, preventing harmful overstimulation of the host immune response [52].

Additional work with several coronaviruses, including severe acute respiratory syndrome coronavirus (SARS-CoV), middle eastern respiratory syndrome coronavirus (MERS-CoV), and the recently emerged severe acute respiratory syndrome coronavirus 2 (SARS-CoV2), has yielded similar findings connecting estrogens and the mediation of severe disease. Several groups have shown that both in animal models and humans, elevated levels of estrogens or ER agonists are associated with improved outcomes of coronavirus infections [53,54,55]. In fact, during the first SARS outbreak, it was reported that being male was significantly associated with intensive care admission or death during infection [56]. During the MERS epidemic and SARS-CoV2 pandemic infected men have been reported to be up to twice as likely to die as women [57,58]. This phenomenon is further corroborated by the fact that premenopausal women, who typically exhibit higher concentrations of estrogens than men, have been reported to be at a lower risk of severe coronavirus infection [55,58,59].

## 4. Influence of Estrogens during Bacterial Infection and Pathogenesis

### 4.1. Estrogens and Their Receptors Regulate Innate Immune Responses to Bacterial Infection

Unlike viruses, bacteria can exist both intra- and extracellularly during infection and thus stimulate a broad array of inflammatory signatures. In general, bacterial pathogens are initially sensed by the host based on their cell wall compositions, which are divided into two major groups based on the terminally exposed structures. Gram-positive bacteria are surrounded by a thick cell wall composed of peptidoglycans, whereas gram-negative bacteria possess a much thinner peptidoglycan cell wall that is encapsulated in an outer lipid membrane comprised of lipopolysaccharides (LPSs). The detection of these different cell wall components is mediated by Toll-like receptors (TLRs). For example, TLR2 in conjunction with TLR1 or TLR6 detects different peptidoglycans, whereas TLR4 detects LPS [60]. These different TLRs induce both a conserved inflammatory response via NF-κB signaling, as well as unique inflammatory signatures that are more tailored to either gram-positive or -negative pathogens [61,62,63,64]. For example, signaling through TLR4 also induces the activation of type-I IFNs, which have been associated with severe inflammatory reactions to gram-negative bacteria [60,65]. 

As mentioned above, estrogens are capable of reducing inflammation by negatively regulating the NF-κB signaling pathway and suppressing IFN signaling [14,16,19]; this has also been observed the context of bacterial-induced inflammation via sensing of molecules such as LPS [66]. Interestingly, it has also been reported that activation of GPER1 is capable of rapidly reducing TLR4 expression in macrophages, identifying an additional ER-associated mechanism of controlling bacterial-induced inflammation [67]. However, this same group also demonstrated that in some instances estrogenes enhance TLR4 expression, suggesting that the regulation of this pathway by estrogens is mediated by the balance of different estrogen receptors across tissues and cell types [68].

### 4.2. Estrogens and Their Receptors Modulate Bacterial Infection Severity

During some bacterial infections, inflammatory responses can progress to sepsis or septic shock, a severe inflammatory state affecting multiple organ systems associated with an up to a 50% mortality rate [69]. Sepsis is typically initiated by bacterially derived molecules that overstimulate recognition receptors, such as TLRs, leading to uncontrolled inflammatory signaling. Gram-negative bacterial infections, for example, can lead to sepsis when pathogenic bacteria actively release LPS, an endotoxin and the most potent stimulator of the TLR4 pathway [70,71]. A number of studies using different sepsis models with altered estrogen levels have reported a generally protective role for estrogens, however the downstream mechanisms are less clear [68,72,73,74]. Notably however, these reports use sepsis models without a true bacterial pathogen, eliminating their ability to evaluate the full impact of estrogen on bacterial infection outcomes. While experimentation with authentic bacterial infection models has been limited, studies suggest that women are up to three times less likely to develop severe sepsis following infection compared to men, potentially indicating protective effect of estrogens [75,76]. Reports of the effects of estrogen administration to cells (or comparisons of cells isolated form men or women) followed by application of TLR agonists have revealed selective effects of the hormone, suppressing some associated responses while enhancing others [77,78,79,80]. The mechanisms underlying these effects may be mediated by affecting TLR expression or the downstream signaling pathways, however more work is required to distinguish between these possibilities.

## 5. Influence of Estrogens during Eukaryotic Infection and Pathogenesis

While less common than bacterial and viral infections, humans are also infected by eukaryotic pathogens, such as parasites and fungi. Eukaryotic pathogens are an extremely diverse group of organisms that vary wildly in size, tissue tropism, pathology, and immune mechanisms of clearance. Here, we will illustrate the impact of estrogens on specific pathogens to illustrate some common themes.

### 5.1. Estrogens and Their Receptors Modulate Parasitic Infection Responses and Severity

Clinically-relevant parasitic pathogens have complex lifecycles that often involve transmission by a vector and require the infection of several hosts, including humans. Malaria is one of the most well-known and common parasitic infections caused by the apicomplexan Plasmodium parasites [81]. These obligate intracellular parasites are transmitted by infected mosquitoes and during their lifecycle transition between infecting red blood cells and liver cells [81]. Interestingly, it has been reported that male hosts sustain up to twice as much parasitic burden and experience up to twice as long infection durations by Plasmodium parasites compared to females, corresponding with enhanced symptom severity [82,83]. During parasitic infections, such as with Plasmodium, it has been established that type-II IFN acts as a critical regulator of the host immune response [84]. Murine models of malaria infection have revealed that administering exogenous estrogen leads to an increase in IFN-γ production, resulting in lower parasitemia and reduced morbidity; accordingly, ovariectomizing mice, thereby reducing estrogen levels, has the opposite effect [85,86]. 

In addition to malaria, other parasitic infections have been found to be influenced by sex, and potentially estrogens. The incidence of amebic liver abscess, which is caused by the protozoan parasite *Entamoeba hysolytica*, has been shown to be up to four times more common in males [87]. These observations were confirmed using animal models, where it was shown that an increase in testosterone, the major male sex hormone, led to a suppression of IFN-γ production and enhanced pathology of the disease [88]. Interestingly, the association between female sex and reduced parasitic infections does not hold true for all parasites. *Toxoplasma gondii* infections, for example, are more common in women, and murine infection models display reduced activation of immune pathways and higher parasitemia and mortality in female mice as opposed to male mice [89,90]. These distinctions in the role of sex and likely impact of estrogens on immune responses during parasitic infection are possibly due to differences in immune signaling molecules and cell types that are required for successful clearance of the pathogen.

### 5.2. Estrogens and Their Receptors Modulate Fungal Infection Responses and Severity

*Candida albicans* is the causative agent of one of the most common fungal infections, vulvovaginal candidiasis (VVC) [91]. Increased levels of estrogens have been shown to be strongly linked with an increased risk of developing VVC in a variety of settings [92,93,94]. One proposed explanation is that *C. albicans* has cellular receptors that can bind estrogen, specifically E2, and this binding promotes growth of the fungal pathogen [95,96,97]. However, recent work has demonstrated that estrogens fail to stimulate the growth of all *C. albicans* strains equally, suggesting that differences in the estrogen binding proteins between fungal species and strains might lead to different outcomes [98]. The growth of *Paracoccidiodes brasiliensi,* the causative agent of paracoccidiodomycosis (PCM), for example, is inhibited by the presence of E2 [99]. Furthermore, unlike *C. albicans* infections, males are at nearly a 10-fold higher risk of developing PCM [100]. In animal models of PCM infection, it has been shown that male mice are at an enhanced risk of severe disease and death, while treatment with estrogen, specifically E2, is capable of reversing this heightened male susceptibility [101]. This is partially explained by the observation that female mice produce enhanced levels of IFN-γ in response to infection by the etiological agent of PCM, *Paracoccidiodes brasiliensis* [102]. Another group found that female mice also demonstrated enhanced survival against *Mucor circinelloides*, one of the causative agents of mucormycosis [103]. The mechanism behind this effect, however, remains unclear and was not consistently seen when looking at a closely related species of Mucor, suggesting estrogens play a more complicated role during fungal infections than in other infectious disease states.

## 6. Effect of Estrogen on Recovery from Infection and Long-Term Immunity

### 6.1. Estrogens and Their Receptors Modulate Recovery after Infection

Following clearance of an invading pathogen, host tissues must begin the process of down-regulating inflammation to prevent excessive damage. This switch is often coupled with the transition of immune cells from inflammatory gene signatures to repair and tissue regeneration signatures, a shift that is critical for the recovery and long-term health of the individual. While many cell types participate in tissue repair following infection, it has been shown that macrophages are key regulators of this process [104]. Their ability to begin coordinating efforts to heal the host after infection is primarily initiated by a shift to a distinct gene profile [105]. During the course of injury/infection, macrophages often display an “M1” profile that is characterized by the production of proinflammatory cytokines and nitric oxide, which aid in the clearance and control of pathogens [106]. During resolution and recovery, however, macrophages shift to an “M2” profile that is instead characterized by the production of the compound ornithine, a key molecule in initiating cell proliferation and other repair processes [107].

Recently, several groups have demonstrated that estrogen, specifically E2, can shift macrophages toward this M2 repair profile in a variety of disease and injury models [17,108,109,110]. Estrogens have been reported to accomplish this primarily through an ERα-dependent pathway that enhances IL-4 pathway activity, a key activator of M2 polarization, along with reducing/blocking the NF-κB signaling and nitric oxide production associated with the M1 proinflammatory state. Contrary to these findings, it has been shown that estrogens reduce tumor size and metastasis in a hepatocellular carcinoma model by blocking M2 polarization in an ERβ-dependent manner [111]. Together these observations suggest the exact effects of estrogen on macrophage polarization are dependent on the ERs present and the microenvironment of the cell. 

Similar observations have been made when looking at estrogen’s effects on T helper cells, which are characterized as having a proinflammatory (Th1) or repair skewed (Th2) gene profile. High levels of estrogen tend to shift T helper cells toward the Th2 transcription profile in an IL-4 related manner, similar to macrophage polarization, an effect frequently observed during pregnancy [112,113]. Considering these findings, it is unsurprising that states of life or being associated with increased concentrations of estrogen, such as women vs. men, pregnancy, and premenopause, are commonly observed as having improved recovery and tissue repair phenotypes [114,115].

### 6.2. Estrogens and Their Receptors Regulate Adaptive Immune Responses to Infection

In addition to affecting innate and early immune pathways, estrogens also affect adaptive immune responses. One way that estrogens influence adaptive immunity is by impacting levels of circulating antibodies. Many groups have demonstrated that estrogen typically correlates with increased levels of antibodies. For example, it has been shown that during enhanced states of estrogen production, such as pregnancy, higher levels of serum antibodies can be detected [116,117]. Similarly, women often exhibit higher antibody responses following vaccination, and this increase in antibodies correlates with better immunity-based protection [118]. These findings have interesting implications in the potential evolution of this predisposition toward enhanced antibody-mediated production during heightened estrogen concentrations. For example, during pregnancy, it is appreciated that strong innate inflammatory signatures, such as type-I IFN signaling, can lead to detrimental outcomes for the developing fetus [14,119]. With estrogen levels at their peak during pregnancy, certain dangerous inflammatory pathways are suppressed, as discussed above, while generation of neutralizing antibodies is enhanced, providing a safer mechanism of immune protection.

Antibodies produced by B cells make up only a part of the adaptive immune response, with T cells representing the other major cell type responsible for our long-term immunity. T cells can be broadly classified into two groups by the presence of specific cell-surface markers, CD8^+^ or “cytotoxic” T cells and CD4^+^ or “helper” T cells. As their names would suggest, these two populations of T cells perform distinct functions during the immune response, with CD8^+^ T cells primarily targeting infected or damaged host cells and inducing cell death to control infection, and CD4^+^ T cells secreting effector molecules (both pro- and anti-inflammatory) to help coordinate the larger immune efforts of other cell types throughout the response [120]. While few studies have been published directly comparing the expression levels of specific ERs in different immune cell types, it is thought that B cells display the highest level of expression of ERs, with an increased ratio of ERβ to ERα; whereas CD4^+^ and CD8^+^ T cells express more intermediate levels, with CD4^+^ cells exhibiting increased levels of ERα [121,122]. Previous work has demonstrated that estrogens are critical for the development of T cells via the use of ER knockout mouse models [121,123]. It has also been shown that estrogens have an anti-inflammatory effect in the context of regulation of helper T cells [41,112,124,125]. These works suggest this is primarily accomplished by blocking NF-κB signaling and shifting helper T cells to their more repair-oriented Th2 signature, as discussed in Section 6.1 [110,112].

## 7. Changes in Estrogen Levels and Impacts on the Immune System

### 7.1. Estrogen Levels during Menstruation, Pregnancy, and Menopause Modulate Immunity

**Figure 2:**
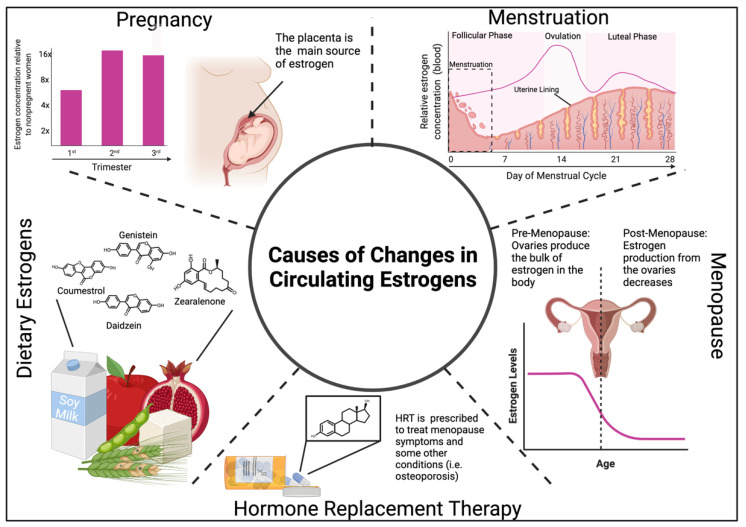
Examples of reasons estrogen levels vary and change. Females experience fluctuating estrogen levels during menstruation, in addition to dramatic changes in hormone levels during pregnancy and following menopause. Exogenous estrogens from dietary or therapeutic (hormonal contraceptives, HRT) sources also alter estrogenic effects in the body. Created with Biorender.com, accessed on 15 January 2022.

When discussing the impacts estrogens have on inflammation and immunity, it is important to consider how and when the levels of these sterols can differ. Prior to menopause, females have approximately 5× as much circulating estrogen as compared to males; however, these concentrations fluctuate with menstrual cycling and can at times be close to that found in males (Figure 2) [126]. Interestingly, females have been documented to experience general low-grade inflammation and an increase in inflammatory markers, such as high sensitivity C-reactive protein, during the start of menstruation when estrogen levels are at their lowest [127,128].

While estrogen concentrations fluctuate during menstrual cycling, these shifts are minor compared to hormone changes during pregnancy. Estrogen levels are often at their highest during pregnancy, peaking during the third trimester when circulating estrogen can be up to 60 times higher than in nonpregnant females (Figure 2) [129]. Given this drastic increase and the immunomodulatory effects of estrogen, it is no surprise that pregnant females have been shown to exhibit altered immune and inflammatory responses. While the changes in the immune system during human pregnancy are complicated by both hormonal and non-hormonal factors, general trends can be observed that align with previously discussed impacts of estrogen on the immune system. For example, pregnancy is associated with a shift toward a Th2 signature, elevated antibody levels, improved autoimmune disease symptoms, and increased expression of key immunomodulatory anti-inflammatory cytokines [112,130,131,132,133].

Estrogen levels in females are lower after menopause (Figure 2) [134]. In general, menopause, and the subsequent reduction in estrogen, is associated with an increase in inflammation and the development of chronic conditions in a variety of organ systems [135,136,137]. While it is difficult to attribute these changes solely to reduced estrogen levels, sudden losses in estrogen due to oophorectomies in premenopausal women can lead to similar outcomes [138,139]. This work also demonstrated that treatment with estrogen therapy can ameliorate the severity of some symptoms. These findings support the specific importance of estrogen to immune health, as opposed the general effects of aging.

### 7.2. Exogenous Estrogens Modulate Immunity

Aside from the natural variations in endogenous estrogen throughout life, individuals can also experience changes in estrogens via exogenous sources. One of the most common sources of exogenous estrogens is hormonal contraceptives, which release high levels of hormones, such as estrogen, to regulate the menstrual cycle and stop ovulation to prevent pregnancy. These increases in circulating estrogen have been shown to impact the immune system and alter inflammatory states. Interestingly, some groups have reported a reduction in inflammation and inflammatory states during hormonal contraceptive use [140,141], while others have reported an increase in inflammatory markers and disease [142,143,144]. These inconsistencies are likely due to a combination of factors, including different routes of contraceptive delivery (i.e., oral vs. vaginal), differing concentrations of estrogen and progestin in contraceptives, and differences in when sampling was performed relative to when contraceptives were taken. 

Another potential source of estrogens is hormone replacement therapy (HRT), where exogenous estrogens are administered to treat a variety of conditions, often postmenopause (Figure 2). Administration of HRT is associated with anti-inflammatory effects and it is commonly used to treat inflammatory diseases in older women [145,146,147]. However, the use of these treatments for some conditions must be tightly regulated as HRT is associated with negative outcomes, such as an increased risk of specific cancers [148]. One of the traditional roles of estrogens is to aid in the development of reproductive tissues, which includes inducing cellular proliferation and growth. This property has been shown to be a major mechanism by which estrogens can drive female-associated cancers (i.e., breast and endometrial cancers) [148,149]. Thus, while the effects of estrogens can lead to improvements in some conditions and even certain types of cancers, it can also stimulate the initiation or recurrence of others.

Humans can also encounter estrogen-like compounds naturally through their diet. For example, phytoestrogens are compounds found in many plants, such as soybeans, that are structurally similar to mammalian estrogen and can even activate ERs (Figure 2). Interestingly, research has demonstrated that diets high in phytoestrogens often lead to similar immune suppression phenotypes as those seen when human estrogen levels are high [150,151,152,153,154]. This immune regulation by phytoestrogens has been suggested by many as a potential treatment mechanism in lieu of the administration of HRT to achieve similar health benefits when estrogen levels naturally decrease, such as in postmenopausal females. Certain fungal metabolites, such as zearalenone, commonly contaminate foods (i.e., grains) and have been shown to have estrogenic effects that similarly regulate the immune system [155]. The specific impact of these compounds, also referred to as mycotoxins, appears to be heavily influenced by the dose, tissues/cell type examined, as well as species of animal studied. For example, it was shown that zearalenone treatment in pigs leads to an increase in several proinflammatory cytokines in the spleen and blood; however, a decrease in these same cytokines was observed in the liver [155,156]. Along these same lines, another group demonstrated that in mice, zearalenone treatment results in reduced proinflammatory signatures in the blood, while the opposite was found when examining the kidneys [157,158]. The complex nature of this interaction is likely attributed to a variety of factors, including differences in the dosing of the mycotoxin, estrogen concentrations in the system, the abundance of specific ERs, as well as differences in how the toxin is processed by the body. Zearalenone, for example, can be processed into two derivatives: alpha- and beta-zearalenol [155]. Together these findings demonstrate the unpredictable immunomodulatory effects of estrogen-like compounds produced by plants and fungi and consumed through diets.

## 8. Conclusions

Traditionally the purpose of estrogens, and other sex hormones, was thought to primarily be the regulation of the development of reproductive organs. It is now apparent however, that these steroids exert a wide range of effects on other systems. In this review, we have discussed some of the commonly observed ways estrogens alter the immune response. In general, it has been shown that estrogens exhibit an anti-inflammatory effect that is associated with improved outcomes during severe infection and wound healing and repair. Differences in the degree to which this observation holds true are likely due to a combination of factors, including the specific pathogen or activator of inflammation, the concentrations of estrogen, estrogenic compounds, and other hormones, as well as the types/abundance of estrogen receptors that are present at the time and location of observation. Continued and future work deciphering hormonal effects on human physiology and immune responses has great potential to explain the heterogeneity in pathogenic responses and disease states across individuals and may facilitate the development of more effective and personalized interventions.
